# Editor's Letter-Box

**Published:** 1898-06-18

**Authors:** 


### Editor's Letter-Box.

[Our Correspondents are reminded that prolixity is a great bar to publication, and that brevity of style and conciseness of statement greatly facilitate early insertion.]

Mr. Walter R. Crofton, of Calmoor Croft, Totton, writes: My attention has been called to an article in The Hospital on "The Inebriates Bill," in which it is stated that "In Ireland a convicts' farm was established at Lusk where inebriates and others who had been frequently convicted were intended to work for their own living, &c. . . . but it had to be abandoned because it was found that the inmates corrupted each other hopelessly." The writer is evidently completely ignorant concerning the object and history of Lusk. This intermediate prison was started by my father, the late Sir Walter Crofton, in 1856, when he was chairman of the Irish Prison Directors. Well-conducted convicts were kept there in huts during the last few months of their sentences reclaiming land, farming, &c. They were in state of semi-freedom, and the object was to gradually fit them for complete freedom. Sir Walter Crofton was convinced of the folly of keeping a man or woman under strict discipline and rules for many years, converting them into machines, and then, without preparation of any sort, turning them loose into the world to do the best they could. "Lusk" was his leading string before perfect liberty. The system and its results were constantly tested and reported on with strong approval by many outside bodies, and in 1879 (after 23 years' trial) a Royal Commission, under Lord Kimberley, recommended the establishment of a similar institution in England, so satisfied were they with the results; and this recommendation was practically repeated by Mr. Herbert Gladstone's recent Committee. Lusk was closed about ten years ago, because there were not enough convicts available in Ireland, the Commissioners making the suggestion specially stating it must not be supposed they were in any way hostile to Lusk. The English officials have always refused to try the system of gradual restoration to liberty and responsibility.

*** The statement respecting the institution at Lusk, which appeared in my paper on "The Inebriates Bill," was quoted from a letter by the secretary of the Howard Association, published in the *Times* a few weeks ago. He had been, I believe, on a tour in Ireland inspecting prisons and reformatories, &c., and I have always found him accurate and trustworthy hitherto. It is clear that he was mistaken as to the reason for closing the establishment at Lusk, but I think it likely that he had heard of difficulties in the administration from the results of free intercourse among the convicts which led him to assign the closure to that cause. The judicious disposal of inebriates is a totally different question from that of convicts generally, but I should be obliged if you would inform your correspondent that I entirely approve of the principle on which the institution at Lusk was founded—the idea of providing an intermediate stage for convicts between prison discipline and absolute freedom is admirable, and it would be well if it could be successfully carried out in the case of all prisoners. I had the pleasure of Sir Walter Crofton's acquaintance in former years, and regret that he should not have spoken to me of his establishment at Lusk.—Contributor.

